# The Diagnostic Pathway of Hirschsprung’s Disease in Paediatric Patients: A Single-Centre Experience

**DOI:** 10.3390/children11080970

**Published:** 2024-08-12

**Authors:** Annita Budzanowski, Niamh Geoghegan, Alexander Macdonald, Muhammad Choudhry

**Affiliations:** Department of Paediatric Surgery, Chelsea and Westminster Hospital, 369 Fulham Road, London SW10 9NH, UK

**Keywords:** Hirschsprung’s disease, rectal biopsy, aganglionosis

## Abstract

Background: The presenting symptoms of patients with Hirschsprung’s disease (HD) are a failure to pass meconium, abdominal distension, and bilious vomiting. The gold standard diagnosis is a rectal biopsy to confirm aganglionosis. The aim of our study was to describe the diagnostic pathway of Hirschsprung’s disease at our institution and document the indication for a rectal biopsy. Methods: We have performed a prospective collection of all patients who underwent a rectal biopsy to exclude HD from December 2022 until September 2023 including. The following data were collected: patient’s age, presenting symptoms, type of biopsy, failure rate, complications, and histopathological results. Results: We identified 33 patients who underwent 34 rectal biopsies at 0.6 years of age. A total of 17 patients had a rectal suction biopsy (RSB), and 17 patients underwent a partial thickness under general anaesthesia (GA). 1/17 (6%) patients had an inconclusive RSB and subsequently underwent a biopsy under GA. Constipation and chronic abdominal distension plus vomiting were the most common presenting symptoms throughout all ages. Five patients (15%) had a rectal biopsy that was positive for HD. Conclusion: A protocolised approach to the assessment of infants and children with suspected HD ensures the appropriate utilisation of invasive procedures such as biopsy.

## 1. Introduction

Hirschsprung’s disease (HD) is a congenital intestinal motility disorder that has a prevalence of 1 in 5000. It is characterised by the absence of ganglion cells in the Meissner and Auerbach plexus in the distal rectum which causes functional distal bowel obstruction [1]. Only a small number of patients (6.4%) present in the first week of life, about 40% in the first 6 months, 50% within the first year, and 93% by 13 years of age [2]. The symptoms are a clinical picture of distal bowel obstruction in the neonatal period with the classic triad of abdominal distension, bilious vomiting, and the delayed passage of meconium, chronic constipation in older children, or enterocolitis [3]. The gold standard diagnosis for Hirschsprung’s disease is a rectal biopsy to confirm aganglionosis in the lamina propria and hypertrophic nerve endings [4]. A rectal suction biopsy (RSB) is a well-established technique first described by Helen Noblett [5] and can be performed at the bedside in the first few months of life. Due to the increasing thickness of the rectal mucosa in older children the success rate of this procedure diminishes and an examination of the ano-rectum under general anaesthesia (GA) and a strip rectal biopsy should be per-formed. Functional constipation in children is a common childhood problem and presents with a prevalence of 5–30% [6]. This large cohort of patients often has complex co-morbidities. A multidisciplinary approach and ongoing joint discussion with the paediatric physician and the incontinence team are important to filter those patients who need further investigations from those who should not be over-investigated. In the United Kingdom, the National Institute for Health and Care Excellence (NICE) published guidance on when to perform a rectal biopsy in children who present with constipation. These include delayed passage of meconium (more than 48 h after birth in term babies), constipation since first the few weeks of life, chronic abdominal distension plus vomiting, a family history of Hirschsprung’s disease, and faltering growth, in addition to any of the previous features (Table 1) [6]. Tan et al. proposed in a critical analysis of rectal biopsy “red flags” for indication for a rectal biopsy, which included the presence of one or more features from the NICE guidance, a history of neonatal distal intestinal obstruction, and a history of genetics known to be associated with HD [7]. The European Reference Network for rare inherited and congenital digestive disorders (ERNICA) recommend a rectal biopsy in the presence of the classic triad of the delayed passage of meconium (>24 h in a term infant), abdominal distension, and bilious vomiting, and in the presence of associated syndromes or a family history of HD. Further, it should be considered in early-onset constipation associated with failure to thrive, in older children with persistent constipation or symptoms of more generalised intestinal motility disorders, and in patients with an absent recto-anal inhibitory reflex (RAIR) on ano-rectal manometry [8]. 

The aim of our study was to describe the diagnostic pathway of Hirschsprung’s disease at our institution and document the indication and presenting symptoms for a rectal biopsy according to the NICE guidance and the incidence of HD in our cohort. 

## 2. Materials and Methods

We performed a prospective data collection (Audit number #WLC11) of all patients who underwent a rectal biopsy to exclude Hirschsprung’s disease at our institution from December 2022 until September 2023 including. 

It is the policy of our institution that all patients who present with lower gastro-intestinal symptoms are under the care of the subspecialist team in paediatric colorectal surgery. The colorectal paediatric surgical team consists of two consultant surgeons, a senior specialist fellow, two paediatric surgical trainees, and two paediatric surgery nurse specialists. The department holds a weekly lower gastro-intestinal multidisciplinary meeting (lower GI MDT) jointly with the paediatric gastroenterology team, which consists of five paediatric gastroenterology consultants and two clinical nurse specialists. Clinical nurse specialists play a crucial role in communication between doctors and parents and are the patient’s advocate. They train parents on rectal washouts, support them with equipment at home, are often the first point of contact when things do not go well, and are aware of the difficulties parents face in daily life. The aim of the joint discussion is to review new or complex patients and their problems from a holistic perspective, ensuring that all team members are being made aware of the patient.

*Rectal washout*

A rectal washout involves the insertion of a catheter into the baby’s rectum and bowel and flushing the bowel with warm saline. The aim is to allow the decompression of the faecal matter, air, and the irrigation fluid. The size of the catheter is dependent on the size of the baby. In our experience, we use size 12 French or 14 French catheter tubes in a term baby. The baby should not be allowed to retain more than 20 mL/kg of fluid [9]. The washouts are performed two to three times per day until adequate decompression is achieved.

All patients who are considered for a rectal biopsy are discussed at the lower GI MDT. 

### 2.1. Diagnostic Pathway in Newborns up to 6 Months of Age

**Figure 1:**
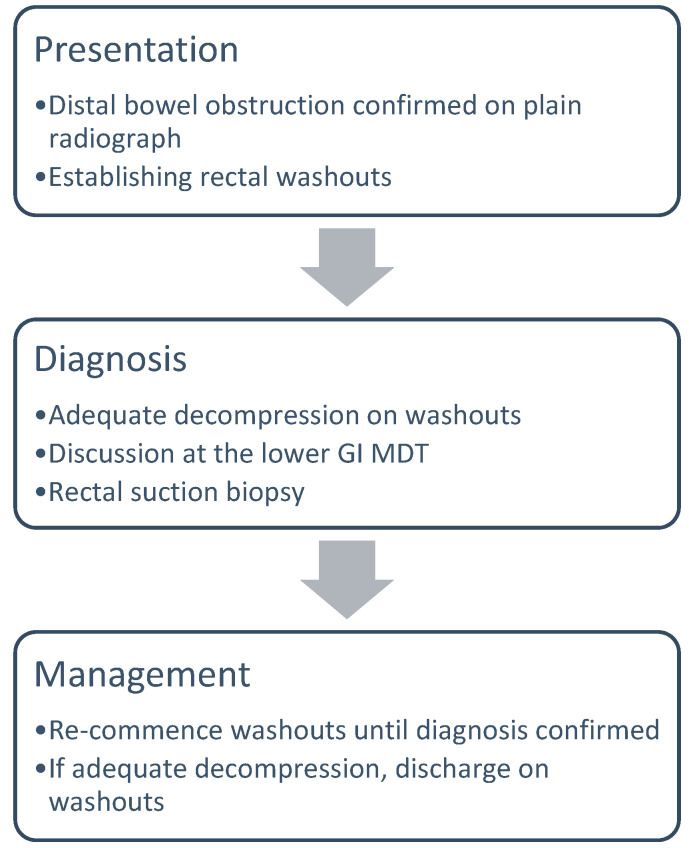
Diagnostic pathway in newborns up to 6 months of age.

A newborn who presents with a clinical picture of distal bowel obstruction, which is demonstrated on a plain radiograph, will be carefully examined, focusing on the exclusion of other differential diagnoses, like ano-rectal malformation. The baby will then be established on rectal washouts aiming to bypass the functional obstruction and achieve equally adequate decompressions of stool and gas. Rectal irrigation is routinely performed with a large bore tube; we preferably use a size 12 or 14 Fr Nelaton Jacques catheter for a term neonate. The catheter is inserted into the rectum and flushed with warmed 0.9% saline, always aiming not to leave more than 20 mls/kg of the baby’s bodyweight inside. This is not only a therapeutic but also a diagnostic manoeuvre, as it aids the exclusion of other differential diagnoses, including meconium ileus or plug and distal bowel atresia. Once an adequate decompression is achieved, a RSB is performed using the RB2^®^ gun (Aus systems, Allenby Gardens, Australia) at the bedside. This procedure is performed without broad-spectrum antibiotic cover as the specimens are very small in size. Rectal washouts are re-commenced 24 h after the biopsy. If satisfactory decompression can be achieved and parents are trained on rectal washouts, the baby is discharged until the diagnosis is confirmed. The assessment of decompression is primarily a clinical assessment focusing on abdominal distension and gastric aspirates. A discussion at the lower GI MDT is imperative prior to rectal biopsy. We do not routinely perform a contrast enema in this group of patients. The indications for a contrast enema are if the baby cannot be decompressed with rectal irrigation or any differential diagnosis other than HD is questioned, like meconium ileus or distal bowel atresia (Figure 1). If the diagnosis of Hirschsprung’s disease is confirmed, then the procedure of choice is a laparoscopic-assisted endo-anal pull-through. 

### 2.2. Diagnostic Pathway in Children Older than 6 Months of Age

A child older than 6 months of age is generally referred with a longstanding history of constipation as well as faecal loading by the general practitioner, paediatric physician, or local emergency department. Some patients will already have had investigations to varying extents. The patient is discussed at the lower GI MDT, aiming for a joint review of presenting history, investigations, and clinical management as per NICE guidance. If the MDT recommendation is to proceed with a rectal biopsy, the child will undergo an examination under GA of the ano-rectum and a partial thickness posterior strip rectal biopsy in the lithotomy position. This procedure is performed with a single dose of a broad-spectrum antibiotic cover, as this is a partial thickness biopsy and the single-size sample is relatively large compared to a suction biopsy. If the diagnosis confirms HD, the colon is washed out under general anaesthesia and a terminal ileostomy is created. In our experience, this group of patients is often very traumatised and has a poor growth velocity. They have been seen by multiple professionals and have had various treatments for their therapy-refractory constipation. Therefore, they will rarely tolerate any rectal washouts while awake and adequate decompression cannot be achieved by oral medication alone. The de-functioned, dilated colon will reduce in size, allowing the child to catch up on their growth and development, aiming for a safe pull-through procedure at the time. 

**Figure 2:**
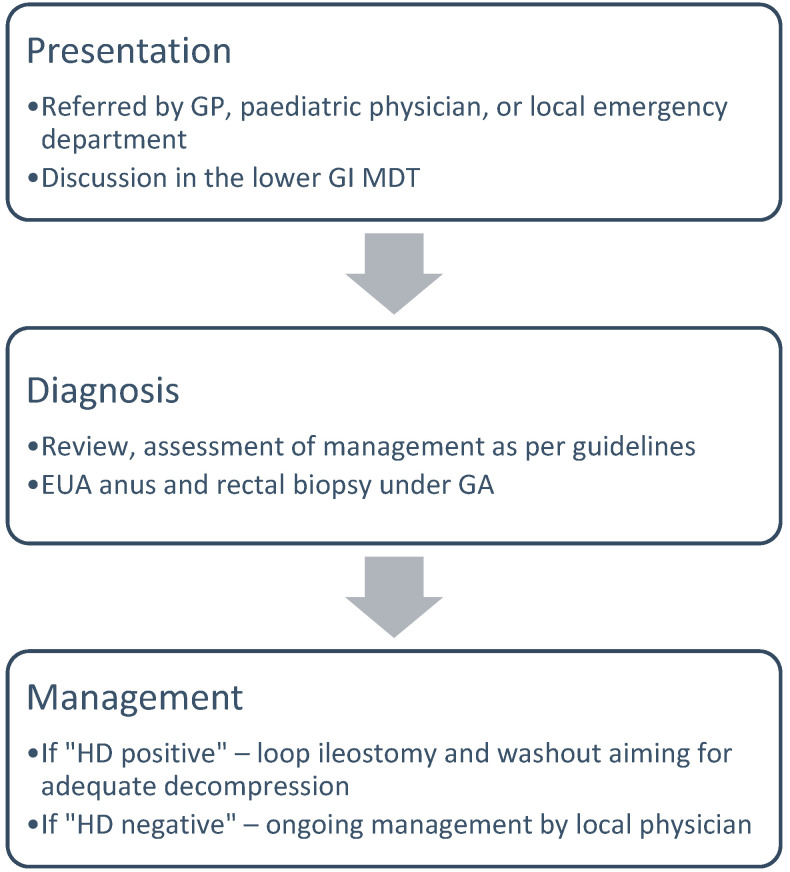
Diagnostic pathway in children older than 6 months of age. EUA—examination under general anaesthesia; GA—general anaesthesia, HD—Hirschsprung’s disease.

**Figure 3:**
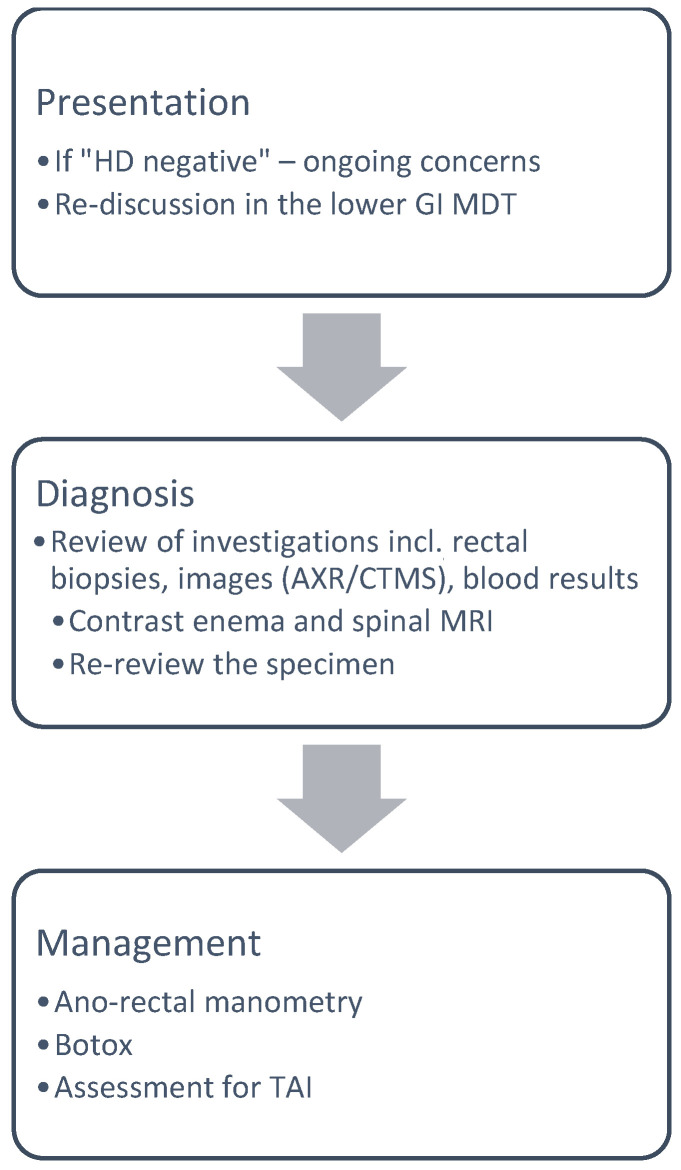
Diagnostic pathway in children if HD-negative.

If the rectal biopsy comes back negative for HD, ongoing care is provided by the local physician (Figure 2). If there are ongoing concerns, the patient is again revisited at the lower GI MDT. All investigations, including radiographs, histopathology results, and blood tests are reviewed. The next investigative steps are magnetic resonance imaging (MRI) of the spine and a contrast enema. The indication for the contrast enema is the assessment of bowel dilatation and exclusion of rare conditions, like functional megacolon or duplication cyst. The indication of the spinal MRI is to exclude spinal dysraphism, including abnormal bony structure, the spinal cord, and the nerve roots. This group of patients is further assessed with functional diagnostics, including ano-rectal manometry, and considered for intra-sphincteric botulinum toxin injection and establishment on transanal irrigation (Figure 3).

All patients who underwent a rectal biopsy for the exclusion of HD during the study period were included. There were no exclusion criteria. The following data were collected: patient’s age, presenting symptoms as per NICE guidance, type of biopsy (strip rectal biopsy or rectal suction biopsy), failure rate, complications, and histopathological results. We used descriptive statistics; data are shown as medians. 

## 3. Results

During the study period, we identified 33 patients (22 male, 65%) who underwent 34 rectal biopsies at a median age of 0.7 years [0.01–12.26]. A total of 17 patients underwent an RSB and 17 patients underwent a partial thickness, posterior strip rectal biopsy and an examination of the ano-rectum under GA. 1/17 (6%) patient had an inconclusive RSB and subsequently underwent a rectal strip biopsy under GA, which was confirmed to be ganglionic. The RSB was performed at 0.2 years [range 0.01–0.69 years]. The rectal biopsy under GA was performed at 4 years [range 0.3–12.3 years]. There were no inconclusive biopsies in the group. We did not encounter any complications in either group. (Table 2) The presenting symptoms and indications for a rectal biopsy as per NICE guidance are listed in Table 3. Constipation since the first few weeks of life and chronic abdominal distension plus vomiting were the overall predominating symptoms throughout all ages. In total, 25 patients (74%) suffered from constipation and 13 patients (38%) had chronic abdominal distension. Only five patients (15%) had a delayed passage of meconium, and one patient had a family history of Hirschsprung’s disease. Faltering growth with any of the other features was encountered in two patients. 

In total, 5/33 patients (15%) had a rectal biopsy that was positive for HD. Four patients had an RSB in the neonatal period and one patient had a delayed diagnosis at 11 years of age. The latter patient underwent an examination of the ano-rectum and rectal biopsy under GA. 2/5 patients (40%) had a delayed passage of meconium and 2/4 patients (40%) had an early onset of constipation. Chronic abdominal distension and vomiting were the predominant symptoms and were identified in 4/5 patients (80%) (Table 4).

## 4. Discussion

This is a single-centre prospective study of all patients who underwent a rectal biopsy for the diagnosis of HD at our institution. HD is a congenital condition that has the predominant feature of distal functional bowel obstructions and presents in the neonatal period with the classic triad of a failure to pass meconium, abdominal distension, and bilious vomiting. Only a small number of patients present in the first weeks of life. About 50% of patients present in the first year of life [2]. There are many factors including presenting symptoms, clinical examination, radiographic images such as abdominal X-ray and contrast enema, and functional diagnostics like ano-rectal manometry that can raise suspicions of Hirschsprung’s disease. The latter one is a time-consuming procedure that requires sedation for younger children and is reliant on the patient’s compliance when they are older. In our practice, we, therefore, do not rely on ano-rectal manometry as a primary diagnostic tool. The gold standard for the diagnosis of Hirschsprung’s disease is a rectal biopsy confirming absent ganglion cells and hypertrophic nerve endings in the plexus submucosus [4].

Various techniques for obtaining a biopsy have been described including surgical, suction, and punch biopsies with many different forceps devices [10,11]. Punch biopsies are, similarly as suction biopsies, performed blindly at the bedside. However, they provide a larger specimen and, therefore, have a higher risk of complication [10,12]. Yoshimaru et al. described a 30-year experience of punch biopsies using an S-moid forceps and a non-specific blood-collecting tube. A total of 954 biopsies were included in this series; 1 (0.1%) patient required a blood transfusion, and 37 (3.9%) patients had an inadequate biopsy obtained. Further, a learning curve and operator experience have been discussed as strong contributing factors for a successful procedure [11]. We agree that this is certainly the case with suction or surgical biopsies in our experience. Alizai et al. observed suction biopsies to be inadequate in children older than 6 months compared with a Storz rectal cup biopsy forceps, being a superior instrument [13]. Some studies described suction biopsy as better indicated for children younger than 36 months of age, due to patient compliance and the presence of a thickened megarectum, which is more likely to lead to an inadequate sample [13,14]. Our experience has been similar; suction biopsies are safe for patients under 6 months of age. However, we acknowledge the need for a case-by-case evaluation offering the best care to the patient and outweighing the risks and benefits with parents. In our cohort, there were three patients who were older than 6 months of age and underwent a suction biopsy at the bedside, the oldest being 8 months old at the time of the procedure. All underwent an uneventful biopsy and were negative for Hirschsprung’s disease. 

There is no single leading presenting symptom for patients with Hirschsprung’s disease. It requires, rather, a careful evaluation of primary symptoms resembling a clinical picture of functional distal bowel obstruction. Patients present in the neonatal period with the classic triad of abdominal distension, bilious vomiting, and the delayed passage of meconium, chronic constipation in older children, or enterocolitis [3]. However, delayed passage of meconium varies between 58% and 90%, abdominal distension in 80% of patients, vomiting in 70% [15], and enterocolitis in 10% to 22% [3,15,16]. In our study, five patients had a rectal biopsy positive for HD. Only 2/5 patients (40%) had a delayed passage of meconium. Two patients (40%) had early-onset constipation and four patients (80%) had a history of abdominal distension and vomiting. None of our patients had enterocolitis as a primary presenting symptom. Clearly, there should be risk stratification and careful decision making for older patients who require a strip biopsy under GA. A procedure under general anaesthesia not only involves greater risk but also has implications for available recourses including theatre capacity, hospital beds, and theatre waiting times, which have been challenging, especially since the COVID-19 pandemic [17]. Lewis et al. [15] recommend that all patients with the delayed passage of meconium, vomiting, and abdominal distension should undergo a rectal biopsy. Nevertheless, there was one patient in their study who had none of these features. Similarly, our department previously published data of 201 patients who underwent a rectal biopsy to exclude HD [7]. The team defined as “*red flags*” the presence of any of the following features: one or more features from the NICE guidance (Table 1), the history of neonatal distal intestinal obstruction, and the history of genetics known to be associated with HD. In total, 43 patients were HD-positive and 7 patients had absent “*red flags*”. All seven patients were above 1 year of age. In our group, none of our patients presented with absent features of NICE guidance. However, careful history taking and clinical examination as well as a multidisciplinary and systematic approach with regular discussions at the lower GI MDT are essential in this group of patients. 

Contrast enema has been described as a helpful adjunct in the diagnosis of HD. Langer recommends that the first step for a baby with a picture of neonatal intestinal obstruction should be a water-soluble contrast enema [3]. The aim is, firstly, to exclude other differential diagnoses, including meconium plug syndrome or meconium ileus, and, secondly, to define the transition zone between aganglionic and ganglionic bowel. The accuracy of the level of the transition zone has been described in about 80% [15,18]. There is also a relatively high false-negative rate of 20–28% in contrast enemas. Therefore, this test should be viewed as supplementary to the rectal biopsy rather than primarily diagnostic [19]. Many surgeons use the contrast enema as a tool to identify a calibre change in the colon and evaluate the level of aganglionosis, and for the subsequent planning of the surgical procedure [4]. A contrast enema is most suitable for evaluating a recto-sigmoid segment but might fail in long-segment HD [20,21]. In our experience, many patients are transferred to our centre and there is often a time delay from presentation to treatment. Further, a number of patients arrive outside of the hours when radiology is not readily available with the given resources. Therefore, we always aim to treat the patient and bowel obstruction first, avoiding worsening of clinical symptoms and development of life-threatening enterocolitis. We use the contrast enema as a diagnostic and therapeutic tool to exclude other rare conditions causing distal bowel obstruction and delineate structural abnormalities of the colon and rectum, like meconium plug syndrome, functional megacolon, or duplication cysts. 

We acknowledge the limitations of our study. Though this is a relatively small number of patients observed over a short period of time, our results are representative when compared with the published literature. The rate of a positive rectal biopsy has been reported between 12% and 17% [13,22]. A review of the rectal suction biopsies demonstrated that the overall risk of RSB is low (0.7%); however, the rate of an inadequate biopsy was at 10% [23]. An open rectal biopsy is considered when the RSB is inconclusive, or in older children when the risk of inadequate biopsy is higher [13]. These results partly mirror our data. Our institution is a regional paediatric surgery unit that covers a catchment area with a population of 2.4 million people in the North West London region. We have a subspecialised lower GI team that primarily looks after this group of patients. A total of 33 patients underwent 34 biopsies over a period of 10 months. One biopsy (3%) was inconclusive and the patient subsequently underwent a repeat biopsy under GA. Five patients (15%) were positive for HD. We did not encounter any complications. The rate of inconclusive RSB at our institution was similar a few years prior at 5% [7]. Rectal suction biopsy using the RB2^®^ gun is a well-described standardised procedure [5]. Though the complication rate is low [23], serious complications, including bleeding, pneumoperitoneum, and death, have been described [24,25]. However, we believe a protocolised approach to the assessment of infants and children with suspected HD ensures that the utilisation of invasive procedures such as biopsy and contrast enema is appropriate and consistent.

## 5. Conclusions

These prospective collected data demonstrate the importance of having a low threshold for a rectal suction biopsy in the neonatal period, which is at a very low risk. A protocolised approach for the assessment of older children who present with constipation and patient-centred care are essential for clinical decisions in patients who present with longstanding constipation. Careful history taking, clinical examinations, and regular discussions in a wider multidisciplinary team are key to identifying those patients who need further invasive investigations such as a rectal biopsy under general anaesthesia. The regular evaluation of problematic patients in specialised teams helps minimise adverse patient outcomes. 

## Figures and Tables

**Table 1:** Indication for rectal biopsy as per NICE guidance.

delayed passage of meconium (more than 48 h after birth in term babies) constipation since the first few weeks of life chronic abdominal distension plus vomiting family history of Hirschsprung’s disease faltering growth in addition to any of the previous features

**Table 2:** Demographics and results and indications as per NICE guidance.

N = 34	RSB N = 17	Rectal Biopsy under GA N = 17
Age (years) [range]	0.2 [0.01–0.69]	4.1 [0.3–12.3]
Gender M/F	12/5	10/7
HD-positive	4 (24%)	1 (6%)
Failure	1 (6%)	0
Complications	0	0

**Table 3:** Symptoms as per NICE guidance.

Symptoms as per NICE Guidance	Total N = 34	RSB N = 17	Rectal Biopsy under GA N = 17
Delayed passage of meconium (>48 h after birth in term babies)	5 (15%)	5 (29%)	0
Constipation since the first few weeks of life	25 (74%)	11 (65%)	14 (82%)
Chronic abdominal distension plus vomiting	13 (38%)	8 (47%)	5 (29%)
Family history of Hirschsprung’s disease	1 (3%)	0	1 (6%)
Faltering growth in addition to any of the previous features	2 (6%)	2 (12%)	0

**Table 4:** Symptoms as per NICE guidance for patients who are HD-positive.

Symptoms as per NICE Guidance	HD-Positive N = 5
Delayed passage of meconium (>48 h after birth in term babies)	2 (40%)
Constipation since the first few weeks of life	2 (40%)
Chronic abdominal distension plus vomiting	4 (80%)
Family history of Hirschsprung’s disease	0
Faltering growth in addition to any of the previous features	0

## Data Availability

Data are contained within the article.
